# Large-Vessel Vasculitis With Autoimmune Myelodysplastic Syndrome: An Uncommon Case of Large-Vessel Vasculitis With Cytopenia That Is Vacuoles, E1 Enzyme, X-linked, Autoinflammatory, Somatic Syndrome (VEXAS) Negative

**DOI:** 10.7759/cureus.103259

**Published:** 2026-02-09

**Authors:** Benjoe George P S, Ann John, Smitha Krishnamoorthy, Caroline Grace Jacob, Saket Chandak

**Affiliations:** 1 Internal Medicine, Amrita Institute of Medical Sciences and Research Centre, Ernakulam, IND

**Keywords:** autoimmune myelodysplastic syndrome, cytopenia, giant cell arteritis-like vasculitis, large vessel vasculitis, vexas-negative

## Abstract

Autoimmune myelodysplastic syndrome (MDS) associated with large-vessel vasculitis (LVV) is an extremely rare clinical presentation. We report the case of an 80-year-old lady presenting with prolonged fever, cytopenias, and inflammatory features, later diagnosed as LVV with autoimmune MDS. Extensive evaluation excluded infectious, malignant, and other autoimmune etiologies, including VEXAS syndrome. The patient responded favorably to corticosteroids and immunosuppressive therapy, showing hematologic and clinical improvement. This case highlights the importance of considering autoimmune bone marrow disorders as a potential cause of unexplained fever with cytopenia and large vessel vasculitis in elderly individuals.

## Introduction

Large-vessel vasculitis (LVV) refers to inflammatory disorders predominantly affecting the aorta and its major branches, typically characterized by granulomatous inflammation. Although these conditions classically involve large arteries, disease expression may be heterogeneous, with variable involvement of branch vessels or medium-sized arteries. The true prevalence of such atypical patterns remains uncertain [1]. Somatic mutations in ubiquitin-like modifier activating enzyme 1 (UBA1), most commonly affecting methionine(Met)at position 41, occur predominantly in myeloid hematopoietic cells. These mutations eliminate the normal cytoplasmic UBA1 isoform and will generate a catalytically impaired Met67 isoform, and give rise to a severe late-onset inflammatory syndrome marked by fevers, cytopenias, marrow vacuolization, neutrophilic inflammation, chondritis, vasculitis, and frequent overlap with conditions such as relapsing polychondritis, Sweet’s syndrome, and myelodysplastic syndromes. Defective ubiquitylation caused by these mutations activates innate immune pathways and drives systemic inflammation, a mechanism that has also been demonstrated in zebrafish models lacking the cytoplasmic UBA1 isoform [2]. Giant cell arteritis predominantly affects individuals over 50 years of age and occurs approximately three times more frequently in women than in men. Glucocorticoids remain the cornerstone of therapy, as they effectively suppress systemic inflammation, normalize inflammatory markers, and reduce the risk of vision loss [3].

Vacuoles, E1 enzyme, X-linked, Autoinflammatory, Somatic (VEXAS) syndrome has recently been recognized as a novel cause of adult-onset autoinflammation accompanied by cytopenias, often mimicking vasculitis or myelodysplastic syndrome [4]. Myelodysplastic syndrome (MDS) comprises a heterogeneous group of clonal hematopoietic stem cell disorders marked by morphological dysplasia in one or more blood cell lineages and persistent peripheral cytopenias, which confer a variable but significant risk of progression to acute myeloid leukemia (AML). The two main subtypes of LVV are giant cell arteritis (GCA) and Takayasu arteritis (TA), both associated with elevated inflammatory markers and systemic features. Autoimmune phenomena are recognized in up to 10%-20% of patients with MDS [5]. MDS and autoimmune disorders (ADs) can coexist or sequentially complicate each other, often with a latency of months to years. MDS-associated ADs show distinct epidemiologic and clinical features but generally do not significantly impact overall survival. Although well documented, this association can be challenging to recognize; therefore, MDS should be systematically considered in patients with ADs who present with unexplained cytopenia [6]. Up to 60% of MDS patients may have serological signs of autoimmunity [7,8]. The survival of MDS patients with or without autoimmune manifestations is generally reported to be similar, indicating that the presence of autoimmune features does not consistently alter overall prognosis. However, some studies have observed that patients with autoimmune manifestations may experience slightly better survival outcomes and a reduced risk of progression to AML, suggesting that autoimmune involvement may influence disease course. [7-9].

This newly identified disease, caused by somatic UBA1 Met 41 mutations, has unified several previously distinct inflammatory and hematologic conditions, each often meeting standard diagnostic criteria, into a single syndrome characterized by a mixed pattern of autoinflammation and autoimmunity. [10]. Vasculitis occurs in approximately one-quarter of patients with VEXAS syndrome, most commonly involving small- and medium-sized cutaneous vessels. Positive antineutrophil cytoplasmic antibody (ANCA) serologies and renal vasculitis may lead to diagnostic confusion. Although cranial manifestations often resemble giant cell arteritis, large-vessel involvement is relatively uncommon in VEXAS syndrome [11].The conventional therapy for LVV involves the administration of glucocorticoids, in some cases co-administered with other immunosuppressive drugs, although the cause of the disease remains unknown. This case describes an elderly woman with LVV and autoimmune MDS but negative for VEXAS mutation, highlighting diagnostic challenges and the overlap between inflammatory and hematologic disorders.

## Case presentation

An 80-year-old woman, with a known history of systemic hypertension, dyslipidaemia, osteoporosis, and valvular heart disease, presented with fever and left molar pain for eight days, followed by generalised weakness and loss of appetite. She was initially treated with oral levofloxacin, following which the molar pain subsided after three days of therapy. However, she subsequently developed a high-grade, intermittent fever associated with chills and loss of appetite. The antibiotic was then switched to cefixime, but there was no clinical improvement.

On admission, she appeared ill and weak. Her vital signs were pulse rate 96 beats per minute, blood pressure 90/70 mmHg, temperature 101°F, and oxygen saturation 98% on room air. Cardiovascular examination revealed a pansystolic murmur best heard in the mitral area, radiating to the left axilla and increasing on expiration, consistent with her known valvular heart disease. Respiratory, abdominal, and neurological examinations were unremarkable. She was admitted for evaluation of fever with bicytopenia. Her initial lab investigation results are presented in Tables 1, 2.

**Table 1:** Laboratory investigations

Parameter	Observed value	Reference range
Procalcitonin (PCT)	0.41 ng/ml	0.05 ng/mL
C-reactive protein (CRP)	179 mg/L	5 mg/L
White blood cell count (WBC)	3.8x10^9 /L	4-11x10^9/L
Neutrophil/lymphocyte ratio (NLR)	74/196	-
Haemoglobin (Hb)	7.35 g/dL	12-16 g/dL
Erythrocyte sedimentation rate (ESR)	139 mm/h	<20 mm/h
Platelet count	294 K/uL	150-400 K/uL
Reticulocyte count	0.94%	0.5-2.5%
Absolute reticulocyte count	19.8x10^9/L	25-100x10^9/L
Sodium (Na)	131.4 mmol/L	135-145 mmol/L
Potassium (K)	3.5 mmol/L	3.5-5.0 mmol/L
Lactate dehydrogenase (LDH)	337 U/L	140-280 U/L
Serum iron	34.7 mcg/dL	60-170 mcg /dL
Ferritin	769ng/mL	15-150 ng/mL
Total iron binding capacity (TIBC)	156 mcg/dL	250-370 mcg/dL
Vitamin B12	636.8 pg/mL	200-900 pg/mL
Thyroid-stimulating hormone (TSH)	2.66 uIU/mL	0.4-4.0 uIU/mL
Vitamin D	28.5 ng/mL	30-100 ng/mL

**Table 2:** Urine routine examination HPF: High-power field.

Parameter	Observed value	Reference range
Pus cells	4-6 HPF	1-5 HPF
Specific Gravity	1.01	1.005-1.030
Urine pH	5.5	4.5-7.8
Red blood cell	2-3 HPF	0-2 HPF
Leukocytes (urine)	Trace	-
Glucose (urine)	Negative	0-56 mg/dl
Granular cast	NIL	0-0
Hyaline cast	NIL	0-0
Epithelial cells	NIL	0-0
Nitrite (urine)	Negative	-
Bacteria (urine)	Absent	0-0
Urine protein	Trace	-
Uric acid crystals	NIL	0-0
Colour (urine)	Colourless	-
Clarity (urine)	Clear	-

**Figure 1:**
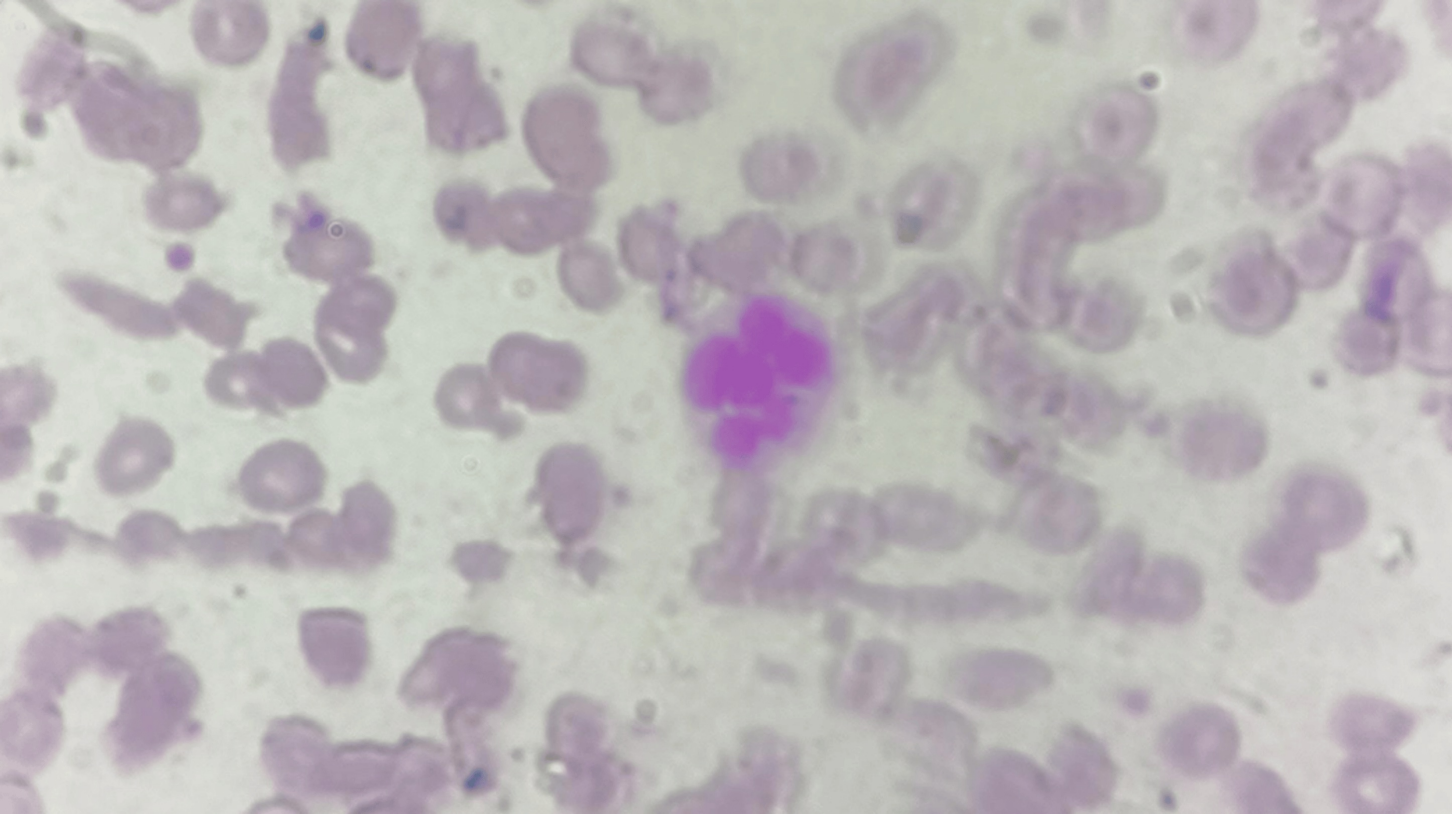
Peripheral smear showing hypersegmented neutrophils and normocytic normochromic anaemia

Laboratory evaluation demonstrated markedly elevated inflammatory markers with a C-reactive protein (CRP) of 179 mg/L and erythrocyte sedimentation rate (ESR) of 139 mm/h as shown in Table 1. Haematological investigations revealed anaemia (haemoglobin 7.3 g/dL) and leukopenia (total leukocyte count 3.8×10⁹/L), suggestive of bicytopenia as shown in Table 1 . Peripheral smear revealed normocytic, normochromic red blood cells with hyper segmented neutrophils and no evidence of hemolysis or blasts as shown in Figure 1. Urine routine examination showed 4-6 pus cells per high-power field as shown in Table 2.

**Figure 2:**
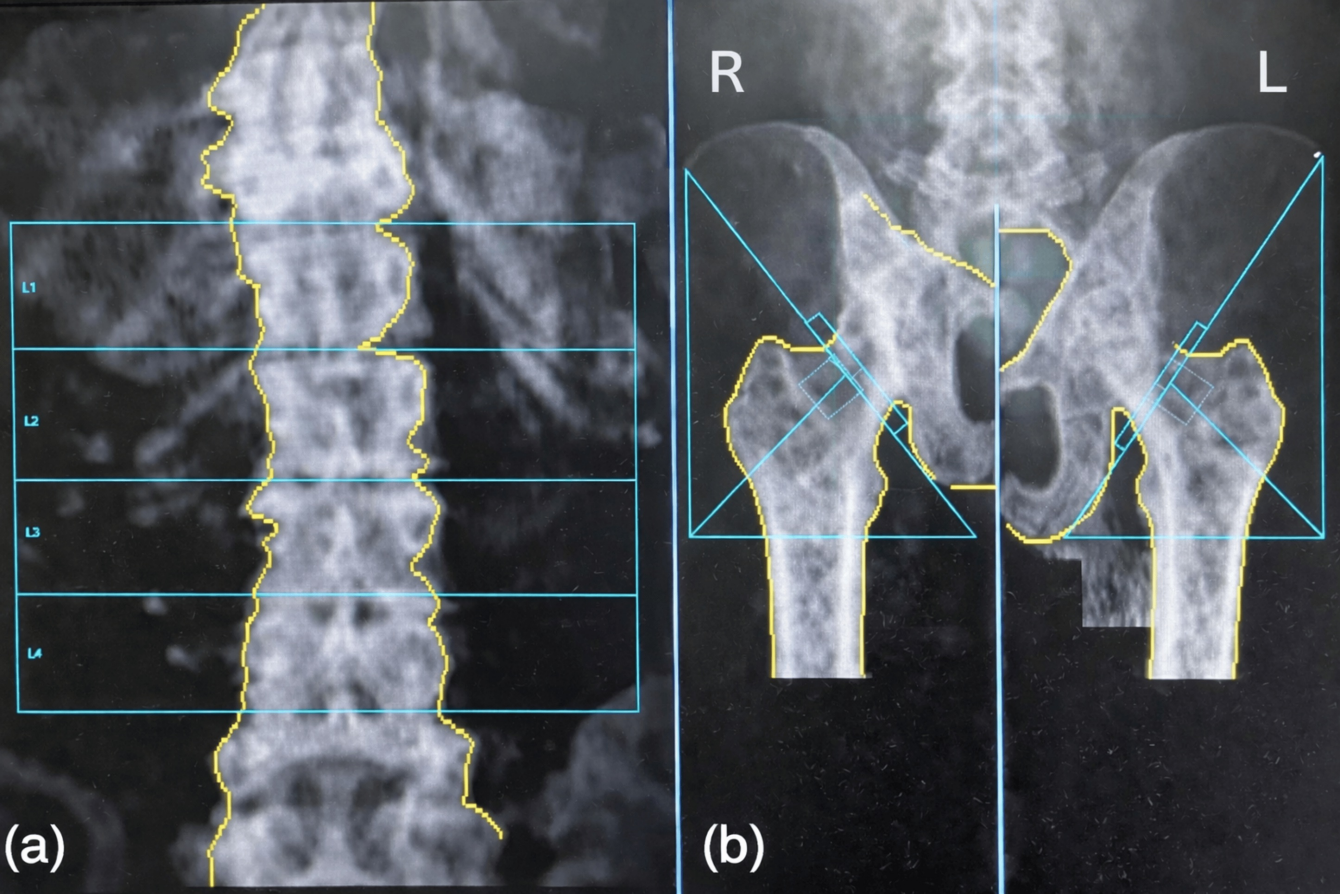
Dual-energy X-ray absorptiometry (DEXA) scan images demonstrating osteoporosis. (a) Anteroposterior lumbar spine (L1–L4) showing reduced bone mineral density with T score of -4.8. (b) Right and left femur showing reduced mineral density over proximal femora with T scores of -2.8 and -3.3, respectively

**Figure 3:**
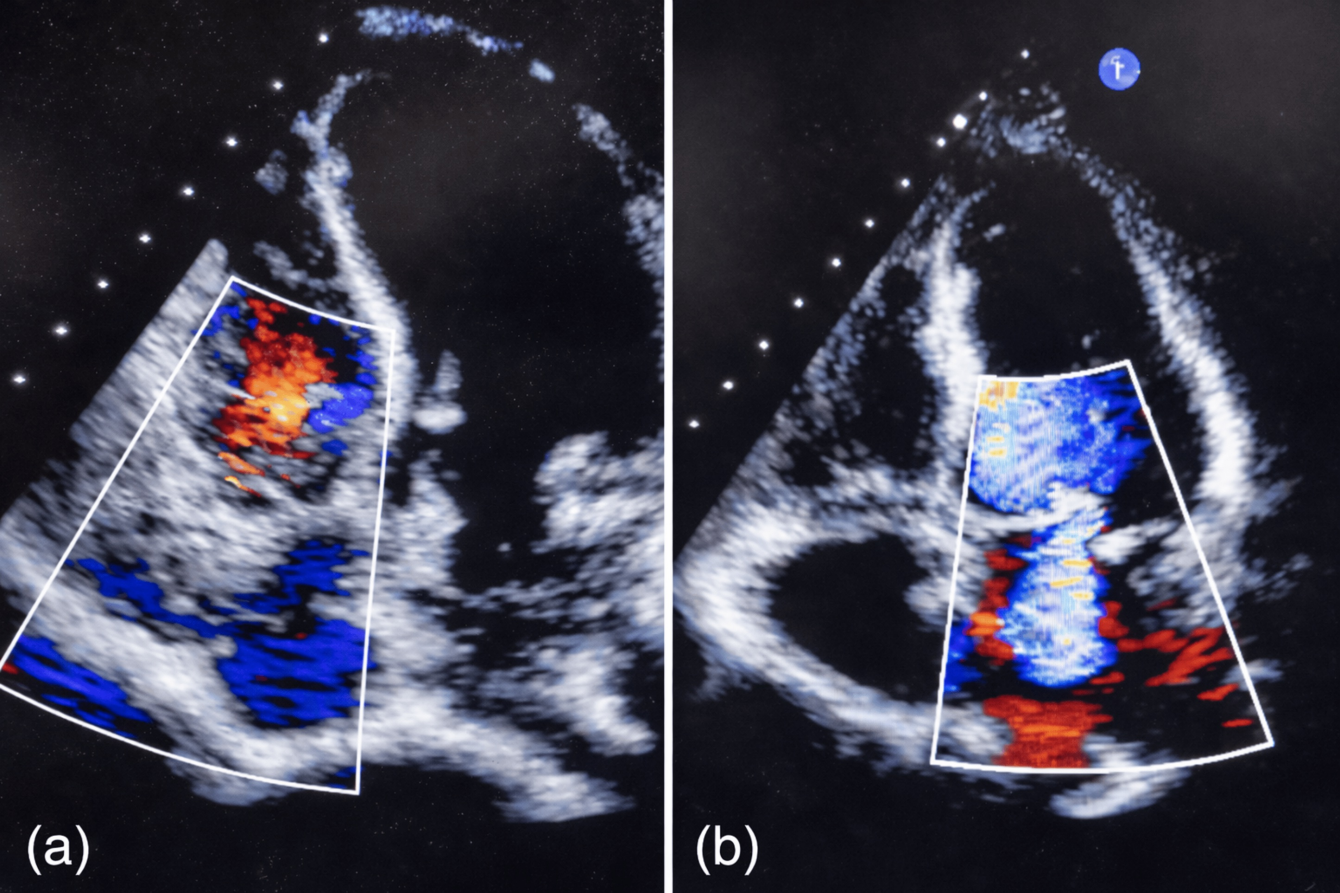
Transthoracic echocardiography with colour doppler imaging demonstrating (a) tricuspid regurgitation and (b) mitral regurgitation, without evidence of valvular vegetations.

She was admitted for further evaluation of fever with bicytopenia. Antihypertensives were withheld due to occult shock. Empirical intravenous piperacillin-tazobactam and teicoplanin were initiated, on the suspicion of infective endocarditis. Blood cultures yielded no growth. A dual-energy X-ray absorptiometry (DEXA) scan revealed severe osteoporosis with T-scores of -4.8 in the anteroposterior (AP) spine, -3.3 in the left femur, and -2.8 in the right femur, as shown in Figure 2. Transthoracic echocardiography (TTE) demonstrated mild to moderate mitral and tricuspid regurgitation without evidence of vegetations, as shown in Figure 3. Antinuclear antibody (ANA) testing by indirect immunofluorescence assay was positive, raising suspicion of an autoimmune vasculitis. ANA blot showed strong positivity for Ro 52 and Sjögren’s syndrome-related antigen A (SS-A), suggesting a strong risk for Sjogren's syndrome as shown in Table 3.

**Table 3:** Antinuclear antibody (ANA) profile showing strongly positive Ro 52 recombinant and Sjögren’s syndrome-related antigen A (SS-A) native antigens

Antigen	Intensity	Class	Interpretation
RNP/Sm	0	0	Negative
Sm	4	0	Negative
SS-A native(60 kDa) (SSA)	97	+++	Strongly positive
Ro-52 recombinant(52)	111	+++	Strongly positive
SS-B(SSB)	4	0	Negative
Scl-70(Scl)	2	0	Negative
PM-Scl100(PM100)	4	0	Negative
Jo-1(Jo)	0	0	Negative
Centromere B(CB)	1	0	Negative
PCNA	1	0	Negative
CsDNA(DNA)	0	0	Negative
Nucleosomes(NUC)	7	0	Negative
Histones(HI)	4	0	Negative
Ribosomal protein(RIB)	1	0	Negative
AMA-M2(M2)	7	0	Negative
Control(Co)	106	+++	Strongly postive
Label(La)	-1		
REFERENCE VALUES			
Intensity	Class	Explanation	
0-7	0	Negative	
8-14	(+)	Borderline	
15-35	+	Positive	
36-70	++	Positive	
71-256	+++	Strongly positive	

**Figure 4:**
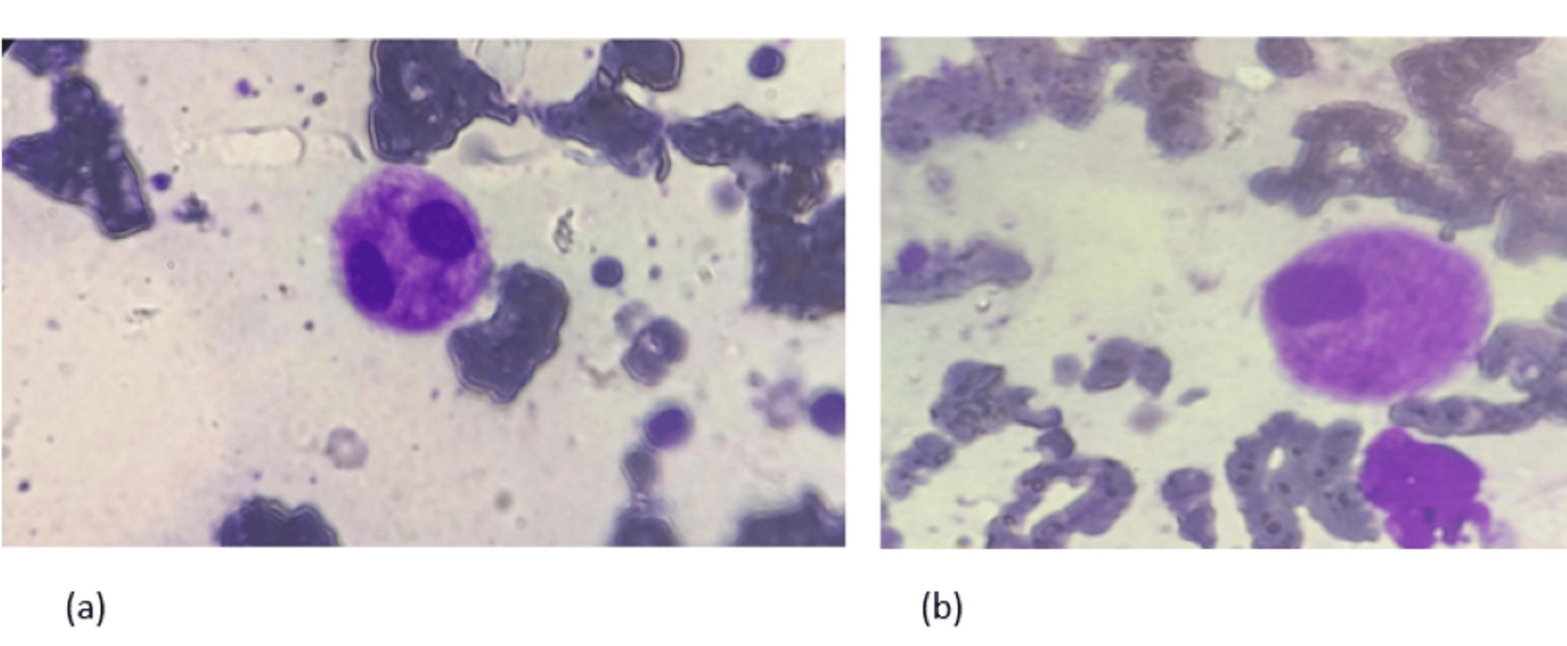
Bone marrow aspirate (a) megakaryocytes with separated lobes. (b) Megakaryocytes with monolobation

Subsequent evaluations showed persistent anaemia and leukopenia (haemoglobin 8.4 g/dL, total leukocyte count 1.8×10⁹/L) along with persistently elevated inflammatory markers (CRP: 26.8 mg/L, ESR: 140 mm/h). Bone marrow aspiration as shown in Figure 4 and biopsy demonstrated trilineage dysplasia with 4% plasma cells and no features suggestive of leukaemia or lymphoma. Karyotyping revealed a normal female chromosomal pattern (46, XX).

A comprehensive leukaemia panel done on peripheral blood revealed the presence of gene transcription factors GATA 2 and U2 AF1 suggesting the strong risk contributed to MDS as shown in Table 4.

**Table 4:** Leukemia panel showing presence of Gene transcription factors GATA 2 and U2 AF1

Gene variant	Allelic function	Function	Classification	Assessment	
GATA2	3.51%(of 114 reads)	Loss	Tier 3	Likely pathogenic	
U2AF1	23.0%(of 667 reads)	Gain	Tier 3	Likely pathogenic	
Result	Positive				

**Figure 5:**
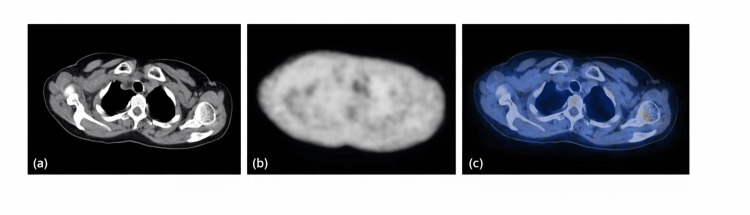
Fluorodeoxyglucose (FDG) PET/CT images at the level of the right subclavian artery. (a) Transaxial computed tomography (CT) image showing the anatomical structures at the thoracic inlet. (b) Corresponding transaxial FDG-PET image demonstrating metabolic activity. (c) Fused transaxial FDG-PET/CT image demonstrating increased FDG uptake along the wall of the right subclavian artery, suggestive of active vascular inflammation.

**Figure 6:**
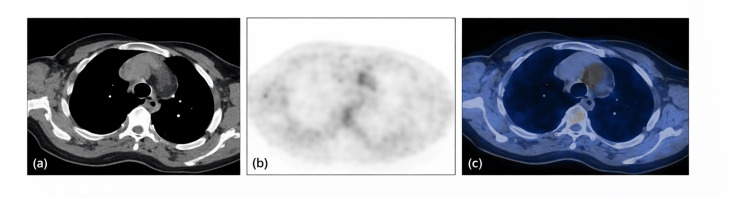
Fluorodeoxyglucose (FDG) PET/CT images at the level of the thoracic aorta. (a) Transaxial CT image depicting the anatomical details of the mediastinum. (b) Corresponding transaxial FDG-PET image showing metabolic tracer distribution. (c) Fused transaxial FDG-PET/CT image demonstrating increased FDG uptake along the aortic wall, consistent with inflammatory involvement of the vessel wall.

A positron emission tomography-computed tomography (PET-CT) scan revealed fluorodeoxyglucose (FDG)-avid wall thickening involving the bilateral subclavian arteries and branches of the aortic arch, findings consistent with LVV as shown in Figures 5, 6. Whole-exome sequencing showed no pathogenic variants in the UBA1 gene, confirming a VEXAS-negative status. The patient was started on oral prednisolone, mycophenolate mofetil, and hydroxychloroquine, along with erythropoietin injections initiated by the haematology team. To treat severe osteoporosis, along with Denosumab injection, synthetic parathormone injections were started. The presence of trilineage dysplasia with cytopenia, normal karyotype, positive autoimmune serology and favourable response to immunosuppression supported the diagnosis of autoimmune myelodysplastic syndrome. Over the following weeks, her haemoglobin and leukocyte count progressively improved, inflammatory markers decreased, and she remained afebrile on follow-up.

## Discussion

The coexistence of LVV and autoimmune MDS is rarely documented, reflecting the complex intersection between haematologic clonal disorders and systemic inflammatory diseases. Although the recent recognition of VEXAS has bridged these entities, this patient was VEXAS-negative, suggesting a separate autoimmune mechanism [4]. This observation underscores the heterogeneity of immune-mediated processes in MDS and the need to distinguish VEXAS from other autoimmune marrow disorders. Autoimmune MDS represents a subset of MDS where dysregulated immune activation contributes not only to cytopenias but also to systemic inflammation, resulting in overlapping haematologic and rheumatologic manifestations [5,6]. In these cases, immune dysregulation may be driven by abnormal T-cell activation, increased release of pro-inflammatory cytokines, and ineffective haematopoiesis, collectively creating a self-perpetuating inflammatory environment. Distinction from VEXAS syndrome is essential, as VEXAS is characterized by UBA1 gene mutations, vacuolated myeloid precursors, and a predilection for older men [4]. These genetic and morphologic hallmarks allow a clear distinction from autoimmune or inflammatory MDS variants that lack UBA1 involvement. VEXAS syndrome, driven by somatic UBA1 mutations, is characterised by systemic inflammation and variable forms of vasculitis; however, large-vessel involvement is relatively uncommon in these patients. Studies suggest small-vessel involvement predominates in VEXAS, while classical LVV-like GCA or Takayasu arteritis, occurs independently and rarely in MDS [12]. Clear recognition of the clinical and morphologic distinctions between these entities is important to avoid both undertreatment and unwarranted exposure to targeted therapies unsuitable for non-VEXAS disease. Vasculitis occurring in MDS patients without UBA1 mutations is uncommon and poses significant diagnostic challenges, necessitating comprehensive clinical, histologic, radiologic, and genetic assessment [13]. Vasculitis has been described in association with MDS, most commonly presenting as leukocytoclastic or other small-vessel vasculitis, while involvement of larger vessels is relatively rare. Behçet’s disease, regarded as a vasculitic disorder, has also been reported in patients with MDS with trisomy 8, particularly among Asian populations [14].

The inflammatory profile in VEXAS often includes fevers, chondritis, pulmonary infiltrates, and cutaneous lesions, with vasculitis most often involving small- to medium-sized vessels. In MDS patients presenting with LVV but lacking UBA1 mutations, alternative diagnoses should be considered, such as GCA, which typically do not exhibit UBA1 mutations [3]. Careful clinicopathologic correlation is therefore required to differentiate between coincidental overlap and pathogenetically related processes. This highlights that although both conditions may share inflammatory pathways, their genetic drivers and histopathologic signatures are distinct. MDS-associated LVV in UBA1-negative patients may mimic classical LVV but often lacks specific features seen in VEXAS, such as neutrophil-predominant infiltrates on biopsy; rather, more typical lymphocyte/macrophage patterns consistent with GCA or Takayasu arteritis may be observed. This distinction carries therapeutic importance, as VEXAS often requires targeted immunomodulation and consideration of haematopoietic-directed therapies, whereas classical LVV responds to conventional vasculitis regimens.

A concise comparison of inflammatory myelodysplastic syndrome, VEXAS syndrome, and classical LVV is provided in Table 5 to highlight key diagnostic and therapeutic distinctions.

**Table 5:** Key Differences Between Inflammatory Myelodysplastic Syndrome, VEXAS Syndrome, and Classical Large Vessel Vasculitis GCA: Giant-cell arteritis; MDS: myelodysplastic syndrome, VEXAS: Vacuoles, E1 Enzyme, X-linked, Autoinflammatory, Somatic syndrome; LVV, large-vessel vasculitis, TA: Takayasu arteritis.

Feature	Inflammatory MDS	VEXAS Syndrome	Classical LVV (GCA/TA)
Typical age	Older adults	Older adults (>50 years)	GCA: >50 years; TA: younger
Sex predominance	No clear predominance	Strong male predominance	Female predominance
Genetic hallmark	No UBA1 mutation	Somatic UBA1 (Met41) mutation	None identified
Bone marrow findings	Dysplasia, autoimmune features	Vacuolated myeloid precursors	Usually normal
Cytopenias	Common	Universal and often severe	Usually absent
Vessel involvement	Rare LVV involvement	Predominantly small to medium vessels	Large vessels (aorta, branches)
Autoimmune serology	Frequently positive	Usually negative	Usually negative
Steroid response	Good	Partial / steroid-dependent	Good
Key references	[5–9,14]	[2,4,10–13]	[1,3]

Management of such overlap syndromes requires multidisciplinary coordination, exclusion of infection, marrow evaluation, immunosuppression, and haematologic support. Integration of rheumatology, haematology, and pathology expertise is critical to determine the underlying mechanism of inflammation and guide treatment decisions. Corticosteroids and mycophenolate provided excellent response in this case, consistent with current therapeutic approaches. This response pattern further supports an autoimmune inflammatory process distinct from VEXAS-related pathology. GCA commonly relapses when glucocorticoids are tapered, and the prolonged use of glucocorticoids is associated with side effects. Treatment with the interleukin-6 receptor-α inhibitor tocilizumab, in combination with glucocorticoids, resulted in sustained remission; however, concerns regarding the safety of tocilizumab remained [3].

Recognition of UBA1-negative MDS with LVV is crucial to prevent diagnostic misclassification and to guide appropriate therapy, as immunosuppressive treatment strategies differ between VEXAS syndrome and classical vasculitis [11]. A systematic diagnostic approach enables accurate classification, informs prognosis, and facilitates individualised therapeutic planning in this complex intersection of haematologic and autoimmune pathology. This case is unique due to coexistence of LVV with autoimmune myelodysplastic syndrome in the absence of UBA1 mutations, highlighting a rare non-VEXAS inflammatory overlap syndrome.

## Conclusions

This case underscores the diagnostic complexity of elderly patients presenting with fever, cytopenia, and vasculitis. When VEXAS syndrome is excluded, autoimmune MDS should be considered in the differential diagnosis. The coexistence of LVV and autoimmune MDS is an exceptionally rare clinical entity, posing significant diagnostic and therapeutic challenges. Although VEXAS syndrome has recently emerged as a unifying diagnosis linking autoinflammation with haematologic abnormalities, this case of VEXAS-negative LVV with autoimmune MDS highlights that alternative autoimmune mechanisms must be considered.

Comprehensive evaluation, including the exclusion of infections, malignancies, and genetic testing for UBA1 mutations, is essential for accurate diagnosis. Histopathologic and radiologic findings may mimic classical LVV such as GCA or Takayasu arteritis; however, the underlying immune dysregulation unique to autoimmune MDS can drive this overlap syndrome. Immunosuppressive therapy with corticosteroids and agents like mycophenolate mofetil can achieve haematologic and clinical improvement in these patients. Awareness and multidisciplinary management are critical to avoid misclassification and optimize patient outcomes in this rare but important intersection of hematologic and autoimmune disease. Timely immunosuppressive therapy can result in significant clinical and haematologic improvement.
